# A Scoping Review of Gut Dysbiosis and Malnutrition in Neurological Disorders: Implications, Indications, and Promising Therapeutic Approaches

**DOI:** 10.3390/jcm15093547

**Published:** 2026-05-06

**Authors:** Thana’ Aljaraedah, Sameeha Al-Thnaibat, Abd Al-Rhman Nawasreh, Wesal Alraei, Esra’a Al-Trad

**Affiliations:** 1 Department of Clinical Nutrition and Dietetics, Faculty of Applied Medical Sciences, Al al-Bayt University, Mafraq 25113, Jordan; dr.wesalalraei@aabu.edu.jo; 2 Department of Nutrition and Integrative Health, Faculty of Allied Medical Sciences, Middle East University, Amman 11831, Jordan; s.thnaibat@meu.edu.jo; 3 Faculty of Medicine, Jordan University of Science and Technology, Irbid 22110, Jordan; nawasreh.abd1@gmail.com; 4 Department of Medical Analysis, Faculty of Applied Medical Sciences, Al al-Bayt University, Mafraq 25113, Jordan; e.ibrahim@aabu.edu.jo

**Keywords:** gut microbiota, malnutrition, neurological disorders, microbiota–gut–brain axis, nutritional interventions

## Abstract

**Background/Objectives**: Neurological diseases are increasing worldwide, but the biological processes underlying these diseases remain poorly understood, and existing treatments have been ineffective at arresting disease progression. Emerging data indicate that dysbiosis of the microbiota–gut–brain axis and malnutrition are comorbid factors in neurological dysfunction. **Methods**: An extended search strategy was developed using a multifaceted approach across various databases to identify eligible studies published between January 2010 and February 2026. **Results**: Results showed uniform relationships among neurological conditions, loss of microbial richness, loss of short-chain fatty acid-producing bacteria, neuroinflammation, and nutritional susceptibility. The review also identifies methodological trends in microbiome profiling and nutritional assessment and suggests an integrative framework of symptom-linked microbial imbalance, malnutrition, and inflammatory processes. **Conclusions**: Although dietary modulation and microbiome-targeted interventions appear promising, the evidence is mostly correlational. Longitudinal and interventional studies should be well-designed to elucidate causal mechanisms and to provide effective clinical strategies.

## 1. Introduction

Neurological disorders pose a significant and increasing societal issue, with a substantial proportion of disability and health burden worldwide. Neurological conditions impact hundreds of millions of people worldwide and are recognised as the top causes of years lived with disability. Their prevalence is on the rise due to population ageing, sedentary lifestyles, and diet-related metabolic risk factors [1,2]. Neurodegenerative and neurodevelopmental diseases, including Alzheimer’s disease (AD), Parkinson’s disease (PD), multiple sclerosis (MS), autism spectrum disorder (ASD), epilepsy, anxiety, depression, and eating disorders, are associated with severe personal, social, and economic burdens. However, the pathogenesis of such diseases remains incompletely understood [3]. Despite improvements in pharmacological and symptomatic treatments, most neurological diseases cannot be cured, and most treatment approaches do not prevent disease progression or target underlying biological drivers [4].

### 1.1. Microbiota–Gut–Brain Axis

Over the past few years, the microbiota–gut–brain axis (MGBA) has emerged as a key mechanism to brain health. The microbiota–gut–brain axis (MGBA) is a two-way communication pathway, which connects the gut microbiota and the central nervous system by neural, immune, endocrine, and metabolic pathways [5,6,7].

### 1.2. Gut Dysbiosis in Neurological Disorders

The clinical and experimental literature consistently demonstrates that gut dysbiosis, i.e., a loss of microbial diversity, depletion of beneficial microorganisms, and increased opportunistic or pro-inflammatory microorganisms, is linked to a wide spectrum of neurological diseases and may contribute to their development, severity, and progression [8,9,10]. Meta-analyses and cross-sectional studies also indicate that autoimmune and neurodegenerative diseases share common dysbiotic characteristics, such as fewer short-chain fatty acid (SCFA)-producing bacteria and more pathogenic taxa, suggesting that microbial signatures underlie distinct phenotypes in the nervous system [11,12].

### 1.3. Malnutrition and Neurological Vulnerability

Not only do undernutrition and diet-induced metabolic imbalance substantially alter the gut microbiome and its activity, but they also directly affect brain structure, neuroinflammation, and cognitive resilience [1,13]. The quality of the diet, fibre intake, body mass index, and metabolic inflammation are closely related to microbial diversity and metabolite production. To illustrate, compliance with healthy, high-fibre diets in PD is associated with increased butyrate-producing, anti-inflammatory microbial populations, which are also linked to high-added-sugar diets [14]. On the other hand, malnutrition and imbalanced nutrition impair the production of microbial-derived neuroactive metabolites, exacerbate systemic inflammation, and further impair gut–brain axis function [9]. Notably, individuals with neurological disorders are especially susceptible to malnutrition because of dysphagia, gastrointestinal dysmotility, cognitive impairment, lack of autonomy, and anorexia caused by the disease, which increases the risk of secondary microbiome-related malnutrition [2,15].

Malnutrition is a multidimensional construct, which includes undernutrition, micronutrient deficiencies, and poor-quality diets, and each of them can affect the microbial diversity, immune regulation, intestinal barrier functioning, and neuroinflammatory processes [13,14].

Micronutrient deficiencies, such as insufficient consumption of different vitamins and trace elements, may affect microbial metabolism, synthesis of neurotransmitters, and immune functioning and, thus, have an impact on neurodevelopmental and neurodegenerative mechanisms [13,14]. Conversely, low-quality diet food, often at the expense of low fibre intake and large amounts of ultra-processed food, has been associated with decreased abundance of anti-inflammatory short-chain fatty acid-producing bacteria and more pro-inflammatory microbial profiles, which is implicated in neuroinflammatory processes [13,14]. Together, these malnutrition dimensions could heterogeneously control the composition and function of the gut microbiota, and subsequently contribute to remaining heterogeneity in neurological outcomes.

Neurological disease, malnutrition, and gut dysbiosis are in self-reinforcing cycles. Pathogenic agents promote poor dietary habits and microbial imbalance. In contrast, neuroinflammation, protein misfolding, and neurodegeneration are stimulated by immune activation, driven by dysbiosis, altered neurotransmitter synthesis, and metabolic impairment [11]. Although the amount of associated data is growing rapidly, causal mechanisms in humans remain poorly understood, and the scope of longitudinal and interventional research is limited [5,16]. This gap is notable given that the gut microbiome is modifiable, and recent evidence indicates that dietary adjustments, probiotics, prebiotics, symbiotics, postbiotics, antibiotics, and faecal microbiota transplantation may positively affect neurological performance [6,17].

### 1.4. Microbiome–Nutrition-Based Therapeutic Approaches

An increasing body of studies suggests that interventions targeting the microbiome and nutrition can therapeutically benefit neurological diseases through well-defined biological pathways along the microbiota–gut–brain axis. Dietary modulation interventions have the potential to change intestinal microbial composition/metabolic output, especially butyrate, a short-chain fatty acid, to improve intestinal barrier integrity and decrease systemic inflammation, microglia activation, and neuronal energy metabolism [2,6]. High-fibre and high-polyphenol diets stimulate the development of anti-inflammatory microbial taxa, whereas malnutrition, deficiencies, and low-quality diets contribute to dysbiosis, impaired neurotransmitter synthesis, and neuroinflammatory pathways [1]. In addition to probiotics, some nutrients have been found to have disease-modifying properties: dietary polyphenols have anti-oxidant and neuromodulatory properties; vitamins (B-complex and vitamin D) regulate neurodevelopment, synaptic plasticity, and immunological tolerance; amino acids (tryptophan and branched-chain amino acids) control neurotransmitter levels and metabolic homeostasis; and prebiotics facilitate microbial fermentation and vagal signalling and consequently cognitive and behavioural functions. In addition, dietary restriction strategies, such as caloric restriction and ketogenic diets, induce metabolic reprogramming, reduced oxidative stress, and microbial community remodelling, thereby promoting neuroprotection and seizure control [17,18,19]. Collectively, the above findings suggest that nutrition-based interventions are not only supportive care but also biologically active, mechanism-based interventions that can modulate disease susceptibility, disease progression, and functional recovery in neurological diseases by re-establishing microbiota balance and metabolic homeostasis.

Recent data also reiterate the clinical importance of microbiota-directed and diet-based intervention in neurological diseases, especially in terms of regulating inflammatory signalling, microbial metabolites, and intestinal barrier functions, which supports the translationalism of the microbiota–nutrition–brain axis.

### 1.5. Scope and Objectives of the Review

The review is a scoping review that visually maps the research literature on the topic, including studies of gut dysbiosis and malnutrition in relation to neurological disorders, as well as methodological trends (microbiome profiling + malnutrition indicators) and knowledge gaps to be addressed in future trials.

This scoping review was based on structured mapping goals rather than causal hypotheses to account for the conceptual breadth of this topic. In particular, the review aimed to answer: what neurological conditions are the most frequently researched in relation to gut dysbiosis and malnutrition; what malnutrition-related indicators and nutritional assessment instruments are used; which microbiome profiling methods are used; what types of dietary or microbiome-specific interventions have been examined and at what evidence levels (animal models, observational studies or randomised trial); and what are the key methodological and translational gaps. With these mapping questions designed to organise the review, the aim is to aid clarity in a heterogeneous body of literature and to outline the priorities of future longitudinal and interventional studies.

### 1.6. Conceptual and Disease-Specific Background

This study concerns the gut microbiota, formerly known as normal flora, a large and complex ecosystem of microorganisms that reside in the human gastrointestinal tract [20]. This microbial community was formerly described as gut microflora, but it is now recognised as crucial in sustaining physiological homeostasis and immune balance [21]. The dominant bacterial phyla of the gut microbiota are Bacteroidetes, Firmicutes, Proteobacteria and Actinobacteria, and Verrucomicrobia and Fusobacteria are less abundant but functionally relevant [22]. Communication between the different commensal microorganisms in the intestinal epithelium is highly dynamic and maintains gut integrity and immune homeostasis in the host [23].

The human gut contains approximately 10–100 trillion microbial cells. Previous estimates indicated that the ratio of microbial cells to human cells could be 10:1, but recent studies have shown that the ratio is close to 1:1 [24]. Despite this diversity, the microbiota is widely recognised as beneficial to host health. Inter-individual and geographic variations also make humans more diverse in their microbiomes, reflecting differences in genetics, diet, environment, and lifestyle [25].

This review also addresses gut dysbiosis, defined as an imbalance in the host microbial ecosystem that disrupts normal physiological processes [5,21]. The microbiota controls immune, metabolic, and neuroendocrine pathways that underpin the microbiota–gut–brain (MGB) axis [2]. Through bottom-up signalling, involving microbial metabolites, the vagus nerve, the enteric nervous system, and systemic circulation, dysbiosis has been associated with a wide range of neurological diseases, such as stroke and Alzheimer’s disease [6].

Studies over the last decade suggest that the gut microbiota is linked to brain development, behaviour, and cognitive function. Metabolites essential for host physiology are encoded by nearly three million microbial genes, far exceeding the 23,000 genes in the human genome [26]. Colonisation begins at birth; some evidence suggests prenatal microbial exposure may influence early-life microbiota development [27]. Neurological consequences may therefore be influenced by disruptions to the microbiota during early development or ageing.

Malnutrition is further considered here in the context of disease-related vulnerability and clinical outcomes. Within this context, malnutrition is a clinically relevant problem that is not well recognised but very widespread among hospital and community groups. Limited clinical data suggest that severe acute malnutrition (SAM) is associated with significant changes in gut microbiota composition. Camara et al. [28] showed in a paediatric cohort that the abundance of *Methanobrevibacter smithii* varied significantly in children with SAM, supporting the idea that malnutrition, in itself, can redefine the gut microbial ecosystem. The results of the studies establish the fact that the lack of microbial balance can be considered not only a characteristic of neurological disease but also a direct result of nutritional deficiency.

In the United Kingdom, malnutrition prevalence among hospitalised patients is estimated at 13–40%; elderly people and those with chronic conditions are especially susceptible to malnutrition [29,30].

According to the European Society of Clinical Nutrition and Metabolism, malnutrition is a condition characterised by insufficient nutrient intake that leads to changes in body composition and physical and mental dysfunction [31]. Neurological disorders are associated with chronic inflammation that can worsen malnutrition by increasing muscle loss and disrupting metabolic regulation, which forms a vicious cycle of cachexia, inflammation, and functional impairment in neurodegenerative diseases [32].

#### 1.6.1. Parkinson’s Disease

Parkinson’s disease (PD) has been repeatedly linked to gut microbiome changes, including reduced Prevotellaceae, increased Enterobacteriaceae, and increased levels of possibly pro-inflammatory taxa, e.g., Enterobacteriaceae [33]. Observational studies indicate that gastrointestinal dysfunction often precedes motor symptoms, and the hypothesis that gut–brain interactions contribute to disease progression is supported. The available human evidence, however, is mostly associative rather than causal. PD may predispose malnutrition because of dysphagia, gastrointestinal dysmotility, and poor appetite, which indirectly affects the microbial composition. Thus, microbial changes have been reported in PD populations, but it is not clear whether dysbiosis is a cause, an adaptation, or a consequence of disease progression. Some of the common causes of malnutrition and weight loss include dysphagia, GIT symptoms, and high expenditure of energy [34]. Taken together, the bidirectional relationships among neurodegeneration, nutritional decline, and microbial imbalance complicate causal inference and underscore the need for longitudinal designs. This impairs the possibility of differentiating whether the presence of microbial changes reflects a main pathogenic mechanism or a secondary effect of disease-related nutritional and gastrointestinal alterations.

#### 1.6.2. Stroke

Animal studies suggest that gut dysbiosis may contribute to the exacerbation of ischemic injury through an immune-mediated process and shifts in inflammatory signalling [35]. The microbial changes and, possibly, the strengthening effects of inflammatory events may also be contributed to by clinical malnutrition that ensues stroke, usually because of dysphagia and excessive metabolic needs. This creates a complex interaction in which neurological injury, immune dysregulation, and nutritional vulnerability may reinforce one another. The reliance on animal models further limits direct clinical generalisability.

#### 1.6.3. Epilepsy

Epilepsy has been linked to changes in gut microbiota and cytokine levels. Experimentally, it was proposed that certain microbial taxa may contribute to seizure vulnerability, and dietary treatment with a ketogenic diet has been observed to alter microbiota composition and seizure rates in animal models [18]. In human research, the evidence remains preliminary and mostly associative. Although the probiotic and dietary approaches have potential, there is a lack of strong randomised controlled studies. Consequently, the translational pathway from microbial modulation to clinical seizure control remains insufficiently defined, particularly regarding causality and clinical responders. Mechanistic interpretation is constrained by the lack of well-powered controlled trials.

#### 1.6.4. Autism Spectrum Disorder

Autism spectrum disorder (ASD) has been associated with gastrointestinal comorbidities and altered microbial composition. There is evidence of a microbial imbalance in the early life stage that may modulate neurodevelopment, yet the causal associations remain under investigation [36,37]. Small-scale research into probiotic and diet-based interventions has mixed results. Recent findings support a modulatory rather than a primary causal role of the microbiome. This suggests that microbial alterations may influence symptom severity or behavioural expression rather than act as a primary cause of the disorder.

#### 1.6.5. Multiple Sclerosis

There is evidence that multiple sclerosis (MS) is linked to immune-mediated changes in gut microbial composition, specifically in Th17 and regulatory T-cell processes. Observational studies report that microbial diversity differs between patients with MS and healthy controls, suggesting that the microbiota may play a role in regulating immunomodulation [38]. Nevertheless, interventional human evidence is scarce, and the bulk of mechanistic understanding is based on experimental models. Additional controlled trials are needed to determine whether microbiome modulation affects disease development. At present, alterations in the microbiota appear more strongly linked to immune regulation than to confirmed disease causation.

#### 1.6.6. Alzheimer’s Disease and Neurodegeneration

Alzheimer’s disease (AD) and amyotrophic lateral sclerosis (ALS) have been associated with alterations in gut microbial diversity and changes in short-chain fatty acid-producing taxa in observational and translational studies [5,9,10,39]. These findings suggest that neuroinflammatory mechanisms, microbial metabolites, and intestinal barrier health may be interrelated. However, most current evidence is cross-sectional, and there is no opportunity to exclude reverse causality, in which a progressive neurodegenerative impairment can modify dietary intake, medication exposures, and gastrointestinal function, thereby restructuring the microbiome. Such results indicate that microbial metabolites, intestinal barrier health, and neuroinflammatory mechanisms may be interconnected. The majority of evidence, however, is cross-sectional, and reverse causality cannot be ruled out: progressive neurodegeneration can modify dietary intake, medication exposure, and gastrointestinal function, thereby remodelling the microbiome. Accordingly, it remains uncertain whether microbial dysbiosis precedes neurodegeneration, accelerates it, or simply reflects systemic consequences of advanced disease.

## 2. Methodology

### 2.1. Study Design and Reporting Guidelines

This study was conducted as a scoping review to map and synthesise the breadth of evidence examining the relationship between gut dysbiosis, malnutrition, and neurological disorders. One reason a scoping methodology was chosen is the breadth of the concept under investigation and the heterogeneity of the study designs, populations, and outcome measures.

The literature was reviewed according to the Preferred Reporting Items for Systematic Reviews and Meta-Analyses extension of Scoping Reviews (PRISMA-ScR). This was aimed at mapping the existing evidence (study designs), defining methodological trends in microbiome profiling and nutritional assessment, and presenting the results in a narrative format to highlight gaps in the research and their translation into the clinical field.

The scoping review methodology was chosen because the study was merely intended to map a rather heterogeneous and expanding literature, as opposed to conducting a very specific and focused quantitative synthesis, unlike a systematic review methodology.

### 2.2. Information Sources and Search Strategy

The literature search was performed in PubMed/MEDLINE, Scopus, and Web of Science with a re-evaluated and optimised search strategy aimed at enhancing the coverage of the literature. The search included articles published between 2010 (January) and 2026 (February). The long time period was chosen to reflect the rapid growth of microbiome sequencing studies and the development of nutrition-oriented neurological research over the last ten years.

The search strategies used a mixture of controlled vocabulary (e.g., MeSH terms in PubMed) and free-text keywording, synonyms, and spelling variations. The Boolean operators (“AND” and “OR”) were used to combine the terms associated with the gut microbiome, dysbiosis, malnutrition, nutritional status, and neurological disorders in order to provide a broad and systematic retrieval of the studies of interest.

Example PubMed Search Strategy: (“gut microbiota” OR “gut microbiome” OR dysbiosis OR “microbiota-gut-brain axis” OR “gut-brain axis”) AND (malnutrition OR undernutrition OR “nutritional status” OR “nutrition assessment” OR “body mass index” OR BMI OR “micronutrient deficiency”) AND (“neurological disorder*” OR “neurodegenerative disease*” OR “Parkinson* disease” OR “Alzheimer* disease” OR epilepsy OR “multiple sclerosis” OR stroke OR “autism spectrum disorder”). Equivalent search adaptations were applied to Scopus and Web of Science using database-specific syntax. 

To be more specific, the number of records retrieved from each database and the subsequent screening steps were documented to enhance transparency. Following the removal of duplicates, all records were screened by titles, abstracts and full-text eligibility. The most prevalent reasons to be excluded from the full-text stage were a lack of microbiome profiling, no specified nutritional assessment, and no neurological outcomes.

The reasons for exclusion most commonly at the full-text stage were: lack of microbiome profiling, research only on microbiome or nutrition and not both, non-neurological, and review/conceptual articles, without primary data. In full-text evaluation, studies were most often removed because of a lack of microbiome profiling, a lack of clearly defined nutritional assessment, emphasis on either microbiome or nutrition alone, a non-neurological outcome, or a lack of primary empirical data.

Furthermore, the relatively small amount of eligible studies can be attributed to the need for simultaneous measurement of microbiome composition, nutritional variables, and neurological outcomes, which is not widespread in the existing literature.

### 2.3. Eligibility Criteria

The eligibility criteria were predetermined to increase the clarity and reproducibility:

- Population: A human subject (of any age) or an animal model of neurological phenotypes.
- Exposure: Evaluation of gut microbiota community by profiling methods (e.g., 16S rRNA sequencing and metagenomic sequencing).
- Nutrition Variable: Incorporation of at least one nutrition-related measure, such as dietary intake measurement, body mass index (BMI), validated malnutrition screening instruments (e.g., MUST and MNA), anthropometric measurements, or nutrition-related biomarkers.
- Outcomes: Diagnosis or characterisation of a neurological disease or neurological phenotype.

The study designs to be included included primary empirical studies, such as randomised controlled trials, cohort studies, case controls, cross-sectional studies, and animal research. Review articles were not considered primary evidence and were consulted on a background level to contextualise and find any other eligible studies while screening references.

Exclusion Criteria: Editorials, commentaries, abstracts of conferences that do not provide full data, studies that do not profile the microbiome, studies that do not provide nutritional assessment, and non-English publications were excluded.

The exclusion of editorials, commentaries, and conference abstracts was due to their overall lack of adequate methodological information and comprehensive data needed to produce credible evidence synthesis.

Inclusion Criteria: Studies were eligible for inclusion if they met the following criteria: (1) involved human participants or animal models with a defined neurological condition or phenotype; (2) included an assessment of gut microbiota composition using recognised profiling methods (e.g., 16S rRNA sequencing or metagenomic analysis); (3) incorporated either direct nutritional assessment (e.g., dietary intake, anthropometric measures, validated screening tools, or biomarkers) or clinically relevant nutrition-related indicators (e.g., gastrointestinal dysfunction or disease-related nutritional risk); and (4) reported outcomes related to neurological disease or function. The core evidence synthesis comprised solely primary empirical studies. These inclusion criteria might be described as restrictive, but they were chosen to be narrow so that there would be conceptual coherence in the microbiome, nutritional, and neurological domains, thus preventing the scattering of the interpretation of isolated variables.

### 2.4. Study Selection Process

The retrieved records were then exported to reference management software for organisation and duplicate removal. Duplicates were detected using automation and confirmed manually. The selection of the studies was done in two steps:

1. Title and abstract screening.
2. Full-text eligibility assessment.

All records were screened by two reviewers individually. The issue of discrepancies was resolved through discussion and consensus. In the event of continued disagreements, a third reviewer was used to introduce objectivity.

**Figure 1:**
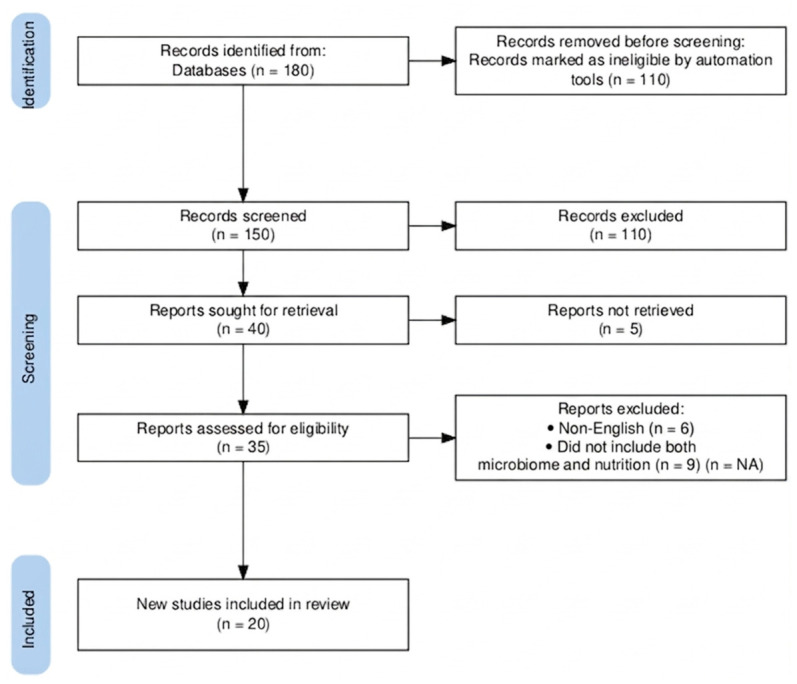
PRISMA-ScR flow diagram illustrating identification, screening, eligibility, and inclusion of studies.

All steps involved in the selection process, including records identified, duplications filtered out, screened records, excluded studies (with reasons), and final inclusions, are presented in the PRISMA-ScR flow diagram (Figure 1).

The PRISMA-ScR flow diagram used in the study selection process is clear and transparent to accomplish its purpose.

### 2.5. Data Charting and Classification

An extracting data-charting form was established before the extraction. The following variables were extracted from each included study:

- Author and year;
- Country;
- Study design.

The characteristics of the population include:

- Neurological disorder;
- Microbiome assessment technique (e.g., 16S and metagenomics);
- Nutrition indicators (BMI, intake, screening tools, biomarkers, etc.);
- Key microbiome findings;
- Key nutrition findings;
- Type of intervention (where appropriate);
- Neurological outcomes.

Two reviewers charted the data and cross-identified them.

The studies were categorised by design (randomised controlled trial, observational human study, animal model, or review article) to differentiate primary empirical evidence from secondary synthesised evidence.

### 2.6. Synthesis of Results

A quantitative meta-analysis could not be conducted due to methodological heterogeneity. A structured narrative synthesis was conducted. Studies were grouped by neurological disorder category, microbiome profiling approach, nutrition assessment method, and intervention type. Evidence was stratified by study design to distinguish between mechanistic findings, associative human data, and interventional research.

This approach supports the objective of mapping evidence patterns rather than establishing causal inference. Considering the heterogeneity of neurological conditions considered, the results were discussed in disease-specific contexts instead of expecting biological similarity between diseases.

## 3. Results

### 3.1. Overview of Findings

The literature generally indicates that malnutrition and gut microbiota imbalance (dysbiosis) are associated with the pathogenesis, progression, and severity of neurological disorders. Alterations in microbial composition, inflammation, and metabolite synthesis are among the most prominent processes linking nutrition, gut health, and brain function.

### 3.2. Literature Review Matrix

Table 1 provides review articles that have been included in the context of interpretation but have not been taken as primary evidence in the synthesis. The results summarised in Table 1 indicate good convergence across the various methodological approaches. Clinical and preclinical evidence supports the consensus that the gut microbiota composition affects neuroinflammation, immune regulation, and disease severity. There are promising therapeutic options in dietary interventions, including probiotics, ketogenic diets, and nutrient supplementation, but their causal relationships are not fully established.

Differences in microbiome profiling approaches and variability in reporting specific microbial taxa limit direct comparability across studies.

The included studies may contribute to the mutual support of the association between dysbiosis, nutritional status and neurological outcome, but the strength and consistency of evidence differ with research designs. Specifically, the differences in microbiome profiling protocols and variations in reporting of individual microbial taxa restrict the possibility of direct comparison and synthesis across studies.

This scoping review highlights the topicality of nutrition and the gut microbiota for neurological well-being. Modifiable risk factors associated with neuroinflammation and metabolic dysfunction across a spectrum of neurological disorders include dysbiosis and malnutrition. Despite evidence suggesting that microbiota-focused and dietary therapies are promising new treatment options, more longitudinal and mechanistic studies are required to establish causality and to inform clinical practice. Nutritional interventions can be combined with traditional treatments to provide a comprehensive approach to reducing neurodegeneration and improving population health outcomes. It is important to keep in mind that not all the included studies directly measured nutritional variables; in some studies, they were estimated using the clinically relevant proxies, including gastrointestinal dysfunction or disease-related nutritional risk, which can influence comparability across studies. Heterogeneity in microbiome profiling techniques, such as non-specific reporting, prevents methodological comparability and emphasises the importance of standardised reporting in subsequent research.

## 4. Discussion

The results of this scoping review provide convergent evidence that neurological disorders are not brain-only conditions but are strongly linked to gut ecology and nutritional conditions via the microbiota–gut–brain axis. The included evidence comprised both primary empirical studies and high-quality reviews, allowing for triangulation of mechanistic insights with clinical observations.

Empirical studies are major sources of information for the synthesis, with the review literature serving as a source of contextual support in interpretation.

These findings are to be carefully interpreted, taking into account the methodological heterogeneity of the studies, which encompasses the variations in microbiome profiling methods among studies, nutritional assessment method variations, and the heterogeneity of study populations. Moreover, confounding variables like patients being exposed to medication, variability in diet, the severity of a disease, and dysfunction of the gastrointestinal system are all possible factors that can independently affect microbiome profiles and preclude the interpretation of any observed evidence as being due to a particular causal mechanism. To this end, the discussion is interested in the interpretation of synthesised evidence and its constraints, as opposed to reiterating prior background concepts.

This association can be clinically significant when assessed in relation to malnutrition, which can manifest as a result of neurological impairment and as an inducer of microbial imbalance and systemic inflammation. These findings, combined with the argument that dysbiosis and malnutrition can reinforce a vicious cycle of neuroinflammation, metabolic dysfunction, and functional decline, were presented in the introductory section [1,2,6]. One key conclusion was that dysbiosis overlaps with immune and inflammatory responses, which can influence neurological outcomes. Microbial metabolites, vagal pathways, enteric networks, and systemic circulation communicate the effects of inflammatory signals to the brain via bottom-up signalling, as illustrated by several studies that describe the microbiota–gut–brain axis as a two-way neuro-immune system [5]. Based on this view, the scoping review suggests that dysbiosis is not merely a marker of disease but may actively contribute to neuroinflammation by producing altered metabolites, modulating immune cell trafficking, and relocating these cells. As the introduction suggests, dysbiotic phenotypes (especially the depletion of SCFA-producing bacteria) are observed across a variety of neurological conditions, further emphasising the role of a trans-diagnostic microbiome in the predisposition to neurological disorders despite disease-specific differences [11,12].

The outcome of a stroke is an especially good predictor of the gut microbiome as a modulator of acute neurologic injury. Wu et al. [35] state that dysbiosis is associated with exacerbation of ischemic damage, and that when the immune system is imbalanced, dysbiosis may influence the degree of brain injury [35]. This is also consistent with Tremlett et al. [8], who raised the issue of immune and inflammatory pathways and a causal relationship between gut dysbiosis and neurological outcomes [8]. This suggests that the gut may act as an immune gatekeeper after stroke, shaping patterns of chemokine signalling and T-cell migration that either exacerbate or suppress neuroinflammation. This is not causal in humans; however, it confirms that strong longitudinal and interventional evidence remains necessary to establish the fact, and it does not refute the use of animal and translational models to provide a mechanistic account [5,16].

The results of Parkinson’s disease also aligned with the previous literature, especially the fact that gastrointestinal malfunction and the microbial differences were related to the disease. Kamperidis & Nightingale found a decrease in Prevotellaceae and an increase in Enterobacteriaceae, which is also confirmation of a change from a taxon that is traditionally associated with fibre fermentation to a taxon with greater inflammatory potential [33]. In the context of diet and malnutrition, dietary malnutrition, gastrointestinal dysmotility, and PD-related dysphagia are suggested to reduce fibre availability and decrease SCFA production, thereby perpetuating inflammation and potentially contributing to neurodegeneration [34]. It aligns with the argument in the introduction that diet patterns can either enrich or deplete anti-inflammatory bacteria, and that susceptibility to malnutrition in neurological disorders may increase damage caused by microbiome disruptions [2,14,15]. Simultaneously, the results of the scoping review are consistent with the warning of the introduction: correlations are significant, yet cause-and-effect relationships in humans require stronger support from prospective designs and controlled interventions [5].

The results from epilepsy studies also provide a new dimension, indicating that the microbiota can alter neuronal excitability through both inflammatory and metabolic mechanisms and can interact with pharmacotherapy. Korf et al. [18] found that higher levels of Lactobacillus and Bifidobacterium are associated with a reduced risk of seizures and that ketogenic diets can alter the microbiota and improve seizure control [18]. This aligns with the introduction’s statement that microbiome-specific approaches and dietary control are potential future treatment options [6,17]. This suggests that the microbiota may affect vulnerability to seizures by producing neuroactive metabolites and immunological cues, and perhaps by altering drug metabolism, a key finding because it implies that the capacity to respond to therapy in epilepsy may depend, in part, on gut ecology. Nonetheless, these results also point to a weakness, namely, the possibility of improvements related to ketogenic diets due to several mechanisms (ketone metabolism, modulation of inflammation, and changes in the microbiota), so it is not always possible to determine the independent role of the microbiome in these cases without highly specific mechanistic studies.

In autism spectrum disorder, the scoping review results are consistent with previous research reporting microbial effects at an early age and gastrointestinal comorbidity. According to the articles by Arrieta et al. [36] and Maguire & Maguire [37], neurodevelopment may be compromised under conditions of early dysbiosis, and the presence of inflammatory microbes, as well as probiotic or prebiotic strategies, may be used to control behaviour and gastrointestinal symptoms [36,37]. This aligns with the introduction’s framing, which claims that disruptions in development can influence cognition, emotion, and behaviour, and that gut inflammation is a common comorbidity in ASD [16]. What this would mean is not that the microbiota is causing ASD, but that there is the possibility of microbiome-mediated immune activation and metabolite signalling being a potentially modifiable risk and symptom amplification layer, particularly in subgroups that have high gastrointestinal symptoms. Such a subtle interpretation is consistent with the introduction’s focus on the complex, multifactorial causation and on improved causal inference in humans [5].

The results of MS also support the current evidence that MS and other immune-mediated neurological diseases are strongly gut-significant. According to Saint-Criq et al. [38], the microbiota influences Th17 and regulatory immune responses, and probiotic interventions can reduce inflammation and disease severity in models and clinical practice [38]. This aligns directly with the introduction, which states that dysbiosis is associated with autoimmune and neurodegenerative diseases and that common microbial perturbations can contribute to various neurological phenotypes [11]. The implication is that MS can be specifically prone to microbiome modulation, as its pathogenesis is centrally mediated. However, the scoping review also suggests that human trials must be particularly cautious about potential confounders, including medication exposure, diet, disability status, and disease duration, as each can independently affect microbial composition.

Reduced abundance of short-chain fatty acid-producing bacteria has been reported in neurodegenerative conditions, including Alzheimer’s disease and amyotrophic lateral sclerosis, in observational and translational studies [5,9,39]. This trend supports the introduction’s focus on diminished SCFA-producing bacteria as a universal dysbiotic characteristic of diseases [11,12]. It is inferred that lower SCFA levels can disrupt gut barrier integrity, augment systemic inflammation, and impair neuroimmune control, further amplifying neurodegenerative cascades, including protein misfolding, oxidative stress, and microglial activation. This finding is also consistent with the broader literature, which shows that immune activation driven by dysbiosis and alterations in metabolite production can accelerate neurodegeneration [39]. Yet, both AD and ALS raise the most compelling concern of reverse causality: the process of neurodegeneration can degrade diet quality and autonomy, increase the risk of malnutrition, and alter medication exposure, all of which may reorganise the microbiome on their own [2,15]. Hence, the evidence supports an association and plausible mechanisms, but does not directly use longitudinal evidence to establish causality.

The most important conclusion from the evidence review is that malnutrition is not merely a background factor but may be a key moderator of microbiota–neurology interactions. The literature suggests that malnutrition is associated with poor outcomes, including reduced fat-free mass, compromised immunity, and increased risk of inflammation and hospitalisation [30,50]. When this is interpreted alongside microbiome evidence, insufficient energy and protein intake, limited fibre intake, and micronutrient deficiencies likely reduce microbial diversity and metabolic benefits, thereby promoting dysbiosis, which, in turn, further increases inflammatory load. This directly supports the introduction of the vicious cycle framework for disease-related impairments, which involves poor dietary intake, microbial imbalance, and dysbiosis-driven immune activation, which hasten neuroinflammation and neurodegeneration [2,11]. Accordingly, the results of the scoping review are consistent with those of Geisler et al. [1]. Gubert et al. [13] found that diet quality and metabolic inflammation are among the biggest drivers of microbial activity and cognitive resilience [13], and this is supported by Kwon et al. [14], who reported that microbial diversity and butyrate levels increase with a healthy, high-fibre diet in PD [14].

Regarding therapeutic implications, the scoping review postulates that therapeutic interventions targeting the microbiome, such as dietary modifications, probiotics, prebiotics, synbiotics, postbiotics, antibiotics, and faecal microbiota transplantation, are functional [6,17].

Recent data further broadened the knowledge base of microbiota-specific nutritional interventions in neurological diseases, especially their role in modulating microbial metabolites, immune regulation, and the gut–brain communication. The recent literature suggests that dietary interventions, such as fibre-based diets, bioactive compounds, and specific nutritional practices, may change the composition and functional products of microbes (in the category of short-chain fatty acids) that are strongly correlated with neuroinflammatory regulation and intestinal barrier maintenance [51,52,53]. Moreover, microbiome-guided nutritional therapies have been linked to the control of neurotransmitter-connected mechanisms and systemic inflammation reactions and it has been proposed that such mechanisms may be relevant in driving disease development and early symptoms. However, the existing body of literature is mainly based on preclinical models and observational human studies, which restricts attributing causation and translating clinical importance. Thus, longitudinal and interventional studies are needed that are designed well to confirm these mechanisms and can be used to establish clinically applicable nutritional approaches to neurological disorders [54,55,56].

Recent data also contribute to the significance of nutrition–microbiota interaction within the microbiota–gut–brain (MGB) axis, which can be described as a two-way communication system between the gut microbiota and the central nervous system via neural, immune, and metabolic pathways.

Collectively, the existing evidence suggests that neurodegenerative processes linked to dysbiosis include disturbance of the gut–brain axis (GBA), augmented gut–brain intestinal permeability, and the synthesis of pro-inflammatory and neuroactive metabolites, including those in kynurenine pathways [41,42]. Nonetheless, causal inference is restricted by the fact that primary evidence is predominated by secondary evidence.

Clinical and biomarker-based effects of nutritional interventions, such as probiotics, prebiotics, and more extensive dietary patterns, have shown encouraging clinical and biomarker-related responses in neurological and neurodevelopmental disorders [42,49]. Certain dietary interventions, including a ketogenic diet (KD) and a Mediterranean diet (MeDi), also seem substantially germane: KD has been linked to seizure management and microbiota restructuring, potentially indicating a microbiome-based mechanism of action [43,45]; compliance with the MeDi has been related to a decreased risk of Alzheimer’s and Parkinson’s diseases, and reduced disease-associated microbial signatures, including reduced short-chain fatty acid-producing taxa and increased pro-inflammatory bacteria in Parkinson’s disease, further support the role of diet–microbiota interactions as potentially modifiable factors in neurological risk [40,44]. Together, these publications reinforce the rising idea of precision, microbiota-targeted nutrition as a preventive and adjunctive approach, yet the causality of this approach is yet to be established by longitudinal and interventional studies [49].

The effects of a ketogenic diet in epilepsy and of diet interventions in cognitive decline indicate that nutritional interventions can produce clinically significant changes and simultaneously alter the microbiota profile [18]. Yet, the fact that the strongest evidence is limited to specific diseases and interventions, and that most findings were obtained from animal models or cross-sectional studies in humans, should be considered in the discussion. This partly aligns with the warning in the introduction that associative evidence grows faster than causal evidence and that well-powered, longitudinal interventional studies remain scarce [5,16]. Hence, although the findings are in line with the direction taken by You et al. [7], who focus on the gut–brain axis as one of the key mechanisms, it is also important to realise that the research will need to be translated into clinical routine once the microbial shifts are linked with neurological biomarkers through standardised microbial measurements and unambiguous respondent subgroups [7].

Notably, causal inference is limited by the preponderance of observational and preclinical evidence. Although mechanistic pathways are biologically plausible, there is currently insufficient evidence to determine directionality, with future longitudinal and interventional research needed to clarify causal relationships.

Generally, the scoping review results are consistent with the literature review, particularly regarding the centrality of the microbiota–gut–brain axis, the dysbiosis–inflammation interaction, the common finding of reduced SCFA-producing taxa across pathologies, and the enhancing effect of malnutrition [2,5,6,11]. The most apparent point of divergence in the findings lies not in direction but in certainty: the evidence reviewed indicates strong associations and mechanisms plausible to humans. Still, it is not sufficiently reliable to establish causality in humans, as noted in the major concerns in the introduction. As a result, dysbiosis and malnutrition should be viewed as treatment goals and clinical factors to be considered in neurological diseases and longitudinal studies, as well as controlled treatments that can clarify the cause, timing, and disease-specific characteristics of microbes; thus, they should be prioritised in future studies.

Causal inferences about the interpretation of findings across disorders should be made with care, as methodological heterogeneity, small sample sizes, and cross-sectional designs limit causal inference. Dysbiosis can, in many cases, be a secondary consequence of disease-induced nutritional and metabolic alterations rather than a primary cause. A longitudinal and intervention study that incorporates standardised microbiome profiling and validated nutritional assessment instruments is needed in the future to define directionality. However, only primary empirical studies were taken into account as the central source of the evidence base for synthesis, and review articles were utilised as the background of interpretation.

### Proposed Integrative Framework and Clinical Translation

The results of this scoping review help justify the need for an integrative systems-level model linking neurological pathology, nutritional vulnerability, and gut microbial changes. According to the evidence that was mapped, we suggest a conceptual model according to which neurological disorders trigger disease-related symptoms like dysphagia, gastrointestinal dysmotility, cognitive impairment, and decreased autonomy. These symptoms predispose to malnutrition by decreasing dietary intake, distorting food choices, and impairing nutrient absorption. In turn, malnutrition facilitates gut dysbiosis, characterised by a loss of microbial diversity, loss of short-chain fatty acid-producing microorganisms, and an increase in pro-inflammatory microorganisms.

**Figure 2:**
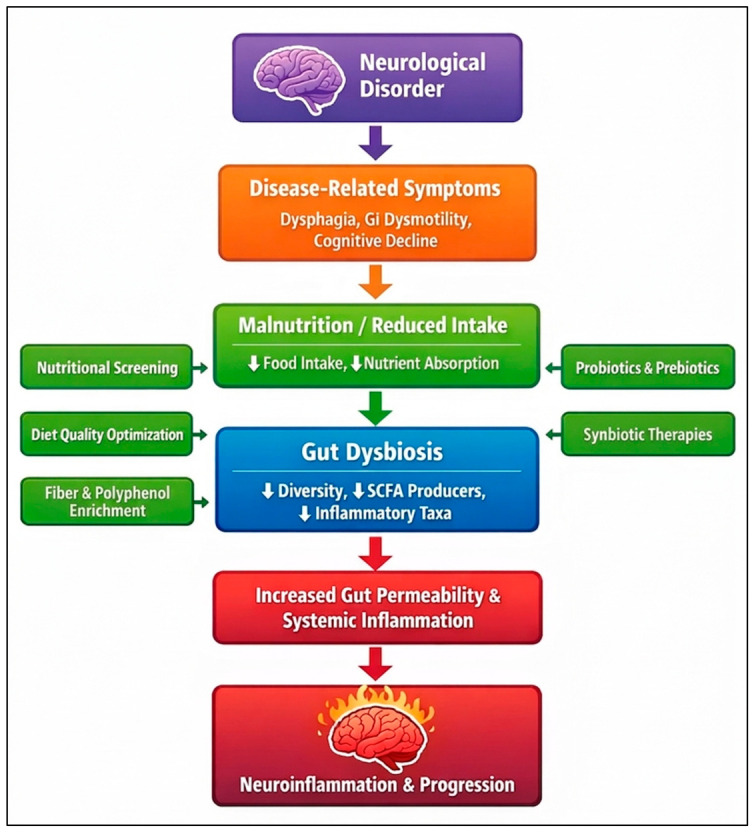
Integrative framework linking neurological disorders, malnutrition, gut dysbiosis, and neuroinflammation with potential clinical intervention points. The downward arrows show disease progression, while the horizontal arrows denote particular points for clinical intervention.

This leads to dysbiosis, which in turn increases intestinal permeability, systemic inflammation, and changes in metabolite production, and activates immune responses within the microbiota–gut–brain axis. These mechanisms increase neuroinflammation, metabolic stress, and pathogenesis, thus strengthening a self-reinforcing loop of neurological impairment, malnutrition, and microbial imbalance. The potential clinical intervention points linking neurological disorders, malnutrition, gut dysbiosis, and neuroinflammation are illustrated in Figure 2.

Notably, this framework offers several clinical entry points. Early nutritional screening, dietary quality assessment, and stratification of malnutrition risk can intervene in the cycle before irreversible changes in microbes occur. Microbial diversity can be restored through specific dietary modulation approaches, such as increased fibre intake, a polyphenol-rich diet, and structured nutritional rehabilitation. Microbiome-specific therapies, including probiotics, prebiotics, synbiotics, or a ketogenic diet, can also be added to metabolic and immunological control in chosen populations. Such interventions should be tailored based on the disease stage, nutritional status, and microbiome phenotype.

This integrative model is not based on a directional cause-and-effect relationship, but rather imagines neurological diseases as a system that dynamically interacts with nutritional and microbial ecosystems. This review is a step toward a more translational approach to the study of the microbiome in disorders and research designs by mapping evidence across both domains, supporting integration of mechanistic insights from microbiome research with routine neurological practice.

A significant value-addition of this review is the stratification of the evidence by methodological level. Most mechanistic findings are based on animal models and the translational literature, which have strong biological plausibility but limited direct applicability to clinical populations. The literature reports that human studies, by design, consistently show a correlation between dysbiosis and malnutrition phenotypes and the severity of the neurological condition. Still, there is no immediate sense of causality. There are relatively few randomised controlled trials that aim to modify diet or the microbiome, and they are usually disease-specific. These levels of evidence mapping help explain the strength of evidence, which is associative but not robust, and the areas with a strong need of high-quality interventional research. This framework constitutes one of the conceptual syntheses based on the mapped evidence instead of a confirmed causal framework.

## 5. Conclusions

This paper aimed to provide a synthesis and critical review of the existing evidence on the combined effects of gut dysbiosis and malnutrition on the aetiology and pathophysiology of key neurological diseases via the microbiota–gut–brain axis. A scoping review was used to achieve this goal, and it was anchored in 20 peer-reviewed articles published between 2010 and 2026. Human clinical trials and animal models were included in these works to examine the gut microbiota composition, dietary status, pathophysiology, and neurological performance. This review was guided by a positivist research philosophy that focused on empirical evidence and a mechanistic interpretation, and by a qualitative thematic synthesis used to synthesise the outcomes of heterogeneous study designs.

Based on the currently available eligible evidence, the key results show a consistent correlation between neurological conditions and dysbiosis of the gut, diminished microbial diversity, loss of beneficial short-chain fatty acid (SCFA)-producing microorganisms, and enrichment of pro-inflammatory or opportunistic microorganisms [2,11]. Immune activation, dysregulation of microbial metabolites, and neuroinflammation were always linked to dysbiosis in varied conditions, such as Parkinson’s, stroke, epilepsy, autism spectrum disorders, MS, Alzheimer’s, and amyotrophic lateral sclerosis [6,28,35]. Interestingly, malnutrition was also identified as a critical but understudied factor that exacerbates microbiome dysbiosis and neurological vulnerability by disrupting immune resilience, reducing microbial diversity, and increasing inflammatory pathways [31,32,50]. This is consistent with prior evidence that diet quality, fibre intake, and metabolic inflammation are inseparably linked to the composition of the microbiota and neurological outcomes [1,14].

The theoretical and practical implications of these findings are significant. Theoretically, the review reinforces the perception that neurological disorders are systemic diseases that affect peripheral biological systems rather than individual brain pathologies. The clinical outcomes indicate the effectiveness of microbiome- and nutrition-based therapies, including dietary modulation strategies, probiotics, prebiotics, and ketogenic or fibre diets, when combined with conventional neurological treatments [6,17,18]. These strategies can minimise neuroinflammation, improve metabolic homeostasis, and enhance quality of life, particularly in high-risk, malnourished patients.

In addition to these contributions, the research has several limitations. To begin with, most studies were observational or preclinical and could not enable cause-and-effect investigations in humans. Second, the lack of methodological homogeneity in measuring the microbiome, diet, and neurological outcomes makes it difficult to directly compare results across studies. Third, the issue of reverse causality cannot be disregarded, as neurological disability in itself can alter diet, pharmacological activity, and even the composition of gut microorganisms [2,15].

Longitudinal and interventional studies are the future research priorities for concurrently measuring nutritional status, microbiome dynamics, and neurological biomarkers. The inclusion of dietary evaluation, improved patient stratification, and standardised microbiome profiling are key to discerning causal pathways and identifying responders to microbiome-targeted treatment [5,16].

The study’s uniqueness lies in treating gut dysbiosis and malnutrition as related factors rather than independent variables in neurological pathology. This analysis of evidence on numerous neurological conditions examines the intersection of microbial and nutritional systems and the viability of a systems perspective on neurological health and disease prevention.

## Figures and Tables

**Table 1:** Overview of research investigating gut microbiota, malnutrition, and neurological consequences.

Author (Year)	Disorder/Focus	Study Design	Population/Model	Sample Size	Microbiome Method	Nutrition Measure	Key Microbiome Finding	Key Nutrition Finding	Intervention Type	Reported Outcome	Limitations
Arrieta et al. [36]	Neurodevelopment/ASD	Review article	Human & animal studies	Not applicable	Various	Early-life nutrition	Early dysbiosis affects development	Diet shapes microbiota	Conceptual	Developmental modulation	Review article; no quantitative synthesis
Camara et al. [28]	Severe acute malnutrition	Observational human	Paediatric cohort	253 participants	Metagenomics	Clinical SAM diagnosis	Altered *M. smithii* abundance	Malnutrition linked to dysbiosis	Nutritional rehab context	Microbiome alteration	Cross-sectional; geographically specific cohort
Gentile et al. [40]	Diet & neurodegeneration	Narrative review	PD & others	Not applicable	16S/meta	Diet patterns	PD dysbiosis signature	Unhealthy diet ↑ risk	Diet	Preventive target	Narrative review; no quantitative synthesis
Holmes et al. [2]	Age-related neurological diseases	Review article	Human & animal studies	Not applicable	Various	Dietary patterns	Dysbiosis linked to neuroinflammation	Nutrition influences microbiota	Conceptual	Therapeutic implications proposed	Review article; no primary data
Kamperidis & Nightingale [33]	Parkinson’s disease	Observational clinical	Adult patients with PD	NR	16S rRNA sequencing	Not directly measured	Reduced Prevotellaceae; ↑ Enterobacteriaceae	GI dysfunction may affect intake	None	Association with severity	Sample size not verified; observational design
Kearns [41]	Gut permeability	Narrative review	Patients & models	Not applicable	–	Diet/lifestyle	Dysbiosis increases permeability	Diet restores balance	Diet/probiotics	Reduced inflammation	Mechanistic review; limited clinical trials
Korf et al. [18]	Epilepsy	Animal model	Female rodents	NR	16S rRNA sequencing	Ketogenic diet	Microbial shifts linked to seizures	KD alters microbiota	KD	Reduced seizure vulnerability	Animal model; sex-specific limitation
Koumpouli et al. [42]	NDDs & GBA	Systematic review	Human patients	NR (multiple studies)	Various	Pre/probiotics	Dysbiosis linked to GBA impairment	Nutrition improves outcomes	Pre/probiotics	Clinical improvement	Heterogeneity across studies
Lim et al. [43]	KD & disorders	Narrative review	Multiple disorders	Not applicable	16S/shotgun	KD	↑ Bacteroidetes/Firmicutes shift	KD therapeutic	KD	Symptom improvement	Narrative synthesis; inconsistent datasets
Lubomski et al. [44]	PD prediction	Cross-sectional	103 PD/81 controls	184 participants	16S rRNA V3–V4	Macronutrients	Altered general profile	Carbohydrate intake predictive	Observational	AUC 0.74	Cross-sectional; potential confounding
Mengoli et al. [45]	Epilepsy & KD	Narrative review	Patients & models	Not applicable	Various	KD	KD alters microbiota	KD therapeutic	KD	Reduced seizures	Narrative review; no meta-analysis
Meyer et al. [46]	Cognitive function	Observational human	Midlife adults	597 participants	16S rRNA sequencing	Dietary patterns	Microbial diversity linked to cognition	Diet associated with microbiome	Observational	Cognitive correlation	Cross-sectional; no causality inference
Mitrea et al. [17]	Neuro & psychiatric	Narrative review	Multiple disorders	Not applicable	–	Probiotics	Dysbiosis across disorders	Probiotics beneficial	Supplementation	Symptom modulation	Limited RCT evidence
Saint-Criq et al. [38]	Ageing inflammation	Review article	Human & animal studies	Not applicable	Various	Malnutrition context	Dysbiosis contributes to inflammation	Malnutrition worsens immunity	Conceptual	Immune dysregulation	Review article; indirect evidence
Solch et al. [47]	MeDi & AD/PD risk	Systematic review	At-risk adults	12 included studies	Pooled microbiome data	Mediterranean diet	Favourable microbial profile	Reduced AD/PD risk	Diet	Risk reduction	Study heterogeneity; observational bias
Sorboni et al. [6]	Neurological disorders	Review article	Human & animal studies	Not applicable	Various sequencing approaches	Diet variables	Microbial metabolites mediate gut–brain axis	Diet modulates signalling	Conceptual	Mechanistic synthesis	Review article; heterogeneous evidence base
Stadlbauer et al. [48]	Dementia	Observational human	Elderly patients	41 participants (23 dementia, 18 controls)	16S rRNA sequencing	Not quantified	Gut barrier dysfunction	Nutrition vulnerability	None	Inflammation–cognition link	Small sample; pilot nature
Tan et al. [39]	AD/PD	Narrative review	Human patients	Not applicable	–	Diet patterns	Dysbiosis precedes deficits	Diet drives dysbiosis	Diet/FMT	Preventive potential	Narrative review; no pooled data
Tuigunov et al. [49]	Precision nutrition	Systematised review	AD, PD, MS	NR	Metagenomics	Bioactive nutrients	Dysbiosis drives pathology	Nutrients modulate the axis	Diet	Preventive potential	Emerging field; limited longitudinal data

Abbreviations: AD, Alzheimer’s disease; ASD, autism spectrum disorder; rRNA, ribosomal RNA; AUC, area under the curve; GBA, gut–brain axis; GI, gastrointestinal; KD, ketogenic diet; MeDi, Mediterranean diet; MS, multiple sclerosis; PD, Parkinson’s disease; NDDs, neurodegenerative diseases; NR, not reported; RCT, randomised controlled trial; SAM, severe acute malnutrition; FMT, faecal microbiota transplantation.

## Data Availability

The original contributions presented in this study are included in the article. Further inquiries can be directed to the corresponding author.

## References

[1] Geisler C., Pankoke J., Schlicht K., Knappe C., Rohmann N., Hartmann K., Settgast U., Türk K., Seoudy A.K., Franke A. (2021). BMI, alcohol consumption, and gut microbiome species richness are related to structural and functional neurological abnormalities. Nutrients.

[2] Holmes A., Finger C., Morales-Scheihing D., Lee J., McCullough L.D. (2020). Gut dysbiosis and age-related neurological diseases: An innovative approach for therapeutic interventions. Transl. Res..

[3] Agarwal U., Paliwal S., Yadav V., Pannu A., Tonk R.K., Verma S. (2025). Pathological insights into neurodegenerative and neurodevelopmental disorders: Perspectives for the development of novel treatment approaches. CNS Neurol. Disord. Drug Targets.

[4] Alkahtani S., Al-Johani N.S., Alarifi S. (2023). Mechanistic insights, treatment paradigms, and clinical progress in neurological disorders: Current and future prospects. Int. J. Mol. Sci..

[5] Cryan J.F., O’Riordan K.J., Sandhu K., Peterson V., Dinan T.G. (2020). The gut microbiome in neurological disorders. Lancet Neurol..

[6] Sorboni S.G., Moghaddam H.S., Jafarzadeh-Esfehani R., Soleimanpour S. (2022). A comprehensive review on the role of the gut microbiome in human neurological disorders. Clin. Microbiol. Rev..

[7] You M., Chen N., Yang Y., Cheng L., He H., Cai Y., Liu Y., Liu H., Hong G. (2024). The gut microbiota–brain axis in neurological disorders. MedComm.

[8] Tremlett H., Bauer K.C., Appel-Cresswell S., Finlay B.B., Waubant E. (2017). The gut microbiome in human neurological disease: A review. Ann. Neurol..

[9] Liu L., Wang H., Chen X., Xie P. (2023). Gut microbiota: A new insight into neurological diseases. Chin. Med. J..

[10] Bicknell B., Liebert A., Borody T., Herkes G., McLachlan C., Kiat H. (2023). Neurodegenerative and neurodevelopmental diseases and the gut–brain axis: The potential of therapeutic targeting of the microbiome. Int. J. Mol. Sci..

[11] Chidambaram S.B., Essa M.M., Rathipriya A.G., Bishir M., Ray B., Mahalakshmi A.M., Tousif A., Sakharkar M.K., Kashyap R.S., Friedland R.P. (2022). Gut dysbiosis, defective autophagy and altered immune responses in neurodegenerative diseases: Tales of a vicious cycle. Pharmacol. Ther..

[12] Deng X., Gong X., Zhou D., Hong Z. (2025). Perturbations in gut microbiota composition in patients with autoimmune neurological diseases: A systematic review and meta-analysis. Front. Immunol..

[13] Gubert C., Kong G., Renoir T., Hannan A.J. (2020). Exercise, diet and stress as modulators of gut microbiota: Implications for neurodegenerative diseases. Neurobiol. Dis..

[14] Kwon D., Zhang K., Paul K.C., Folle A.D., Del Rosario I., Jacobs J.P., Keener A.M., Bronstein J.M., Ritz B. (2024). Diet and the gut microbiome in patients with Parkinson’s disease. npj Park. Dis..

[15] Vaia Y., Bruschi F., Tagi V.M., Tosi M., Montanari C., Zuccotti G., Tonduti D., Verduci E. (2024). Microbiota gut–brain axis: Implications for pediatric-onset leukodystrophies. Front. Nutr..

[16] Tiwari P., Dwivedi R., Bansal M., Tripathi M., Dada R. (2023). Role of gut microbiota in neurological disorders and its therapeutic significance. J. Clin. Med..

[17] Mitrea L., Nemeș S.A., Szabó K., Teleky B.E., Vodnar D.C. (2022). Gut imbalance imbalances the brain: A review of gut microbiota association with neurological and psychiatric disorders. Front. Med..

[18] Korf J.M., Ganesh B.P., McCullough L.D. (2022). Gut dysbiosis and age-related neurological diseases in females. Neurobiol. Dis..

[19] Mattson M.P., Arumugam T.V. (2018). Hallmarks of brain aging: Adaptive and pathological modification by metabolic states. Cell Metab..

[20] Salah A.N., Elleboudy N.S., El-Housseiny G.S., Yassien M.A. (2021). Cloning and sequencing of lsaE efflux pump gene from MDR Enterococci and its role in erythromycin resistance. Infect. Genet. Evol..

[21] Valdes A.M., Walter J., Segal E., Spector T.D. (2018). Role of the gut microbiota in nutrition and health. BMJ.

[22] Das K., Upadhyay S., Oli S. (2023). Modulation of intestinal microbiome: Promising therapies in the treatment of inflammatory bowel disease. Recent Developments in Anti-Inflammatory Therapy.

[23] Carbone E.A., D’Amato P., Vicchio G., De Fazio P., Segura-Garcia C. (2021). A systematic review on the role of microbiota in the pathogenesis and treatment of eating disorders. Eur. Psychiatry.

[24] Saeed N.K., Al-Beltagi M., Bediwy A.S., El-Sawaf Y., Toema O. (2022). Gut microbiota in various childhood disorders: Implication and indications. World J. Gastroenterol..

[25] Wang J.Z., Du W.T., Xu Y.L., Cheng S.Z., Liu Z.J. (2017). Gut microbiome-based medical methodologies for early-stage disease prevention. Microb. Pathog..

[26] Davies C., Bergman J., Eshraghi A.A., Mittal R., Eshraghi R.S. (2022). The gut microbiome: Potential clinical applications in disease management. Gut–Brain Connection, Myth or Reality? Role of the Microbiome in Health and Disease.

[27] Mao X.-Y., Yin X.-X., Guan Q.-W., Xia Q.-X., Yang N., Zhou H.-H., Liu Z.-Q., Jin W.-L. (2021). Dietary nutrition for neurological disease therapy: Current status and future directions. Pharmacol. Ther..

[28] Camara A., Konate S., Alou M.T., Kodio A., Togo A.H., Cortaredona S., Henrissat B., Thera M.A., Doumbo O.K., Raoult D. (2021). Clinical evidence of the role of *Methanobrevibacter smithii* in severe acute malnutrition. Sci. Rep..

[29] Saunders J., Smith T. (2010). Malnutrition: Causes and consequences. Clin. Med..

[30] El-Sayed A., Aleya L., Kamel M. (2021). Microbiota and epigenetics: Promising therapeutic approaches?. Environ. Sci. Pollut. Res..

[31] Principi N., Esposito S. (2016). Gut microbiota and central nervous system development. J. Infect..

[32] Oriá R.B., Freitas R.S., Roque C.R., Nascimento J.C.R., Silva A.P., Malva J.O., Guerrant R.L., Vitek M.P. (2023). ApoE mimetic peptides to improve the vicious cycle of malnutrition and enteric infections by targeting the intestinal and blood-brain barriers. Pharmaceutics.

[33] Kamperidis N., Nightingale J. (2022). Neurological disorders and small bowel dysmotility. Curr. Opin. Gastroenterol..

[34] Panelli S., Calcaterra V., Verduci E., Comandatore F., Pelizzo G., Borghi E., Bandi C., Zuccotti G. (2022). Dysbiosis in children with neurological impairment and long-term enteral nutrition. Front. Nutr..

[35] Wu W., Kong Q., Tian P., Zhai Q., Wang G., Liu X., Zhao J., Zhang H., Lee Y.K., Chen W. (2020). Targeting gut microbiota dysbiosis: Potential intervention strategies for neurological disorders. Engineering.

[36] Arrieta M.C., Stiemsma L.T., Amenyogbe N., Brown E.M., Finlay B.B. (2014). The intestinal microbiome in early life: Health and disease. Front. Immunol..

[37] Maguire M., Maguire G. (2019). Gut dysbiosis, leaky gut, and intestinal epithelial proliferation in neurological disorders: Towards the development of a new therapeutic using amino acids, prebiotics, probiotics, and postbiotics. Rev. Neurosci..

[38] Saint-Criq V., Lugo-Villarino G., Thomas M. (2021). Dysbiosis, malnutrition and enhanced gut–lung axis contribute to age-related respiratory diseases. Ageing Res. Rev..

[39] Tan L.Y., Yeo X.Y., Bae H.G., Lee D.P.S., Ho R.C., Kim J.E., Jung S. (2021). Association of gut microbiome dysbiosis with neurodegeneration: Can gut microbe-modifying diet prevent or alleviate the symptoms of neurodegenerative diseases?. Life.

[40] Gentile F., Doneddu P.E., Riva N., Nobile-Orazio E., Quattrini A. (2020). Diet, microbiota and brain health: Unraveling the network intersecting metabolism and neurodegeneration. Int. J. Mol. Sci..

[41] Kearns R. (2024). Gut–brain axis and neuroinflammation: The role of gut permeability and the kynurenine pathway in neurological disorders. Cell. Mol. Neurobiol..

[42] Koumpouli D., Koumpouli V., Koutelidakis A.E. (2025). The gut–brain axis and neurodegenerative diseases: The role of nutritional interventions targeting the gut microbiome—A systematic review. Appl. Sci..

[43] Lim J.M., Letchumanan V., Tan L.T.H., Hong K.W., Wong S.H., Ab Mutalib N.S., Law J.W.F. (2022). Ketogenic diet: A dietary intervention via gut microbiome modulation for the treatment of neurological and nutritional disorders (a narrative review). Nutrients.

[44] Lubomski M., Xu X., Holmes A.J., Muller S., Yang J.Y., Davis R.L., Sue C.M. (2022). Nutritional intake and gut microbiome composition predict Parkinson’s disease. Front. Aging Neurosci..

[45] Mengoli M., Conti G., Fabbrini M., Candela M., Brigidi P., Turroni S., Barone M. (2023). Microbiota-gut-brain axis and ketogenic diet: How close are we to tackling epilepsy?. Microbiome Res. Rep..

[46] Meyer K., Lulla A., Debroy K., Shikany J.M., Yaffe K., Meirelles O., Launer L.J. (2022). Association of the gut microbiota with cognitive function in midlife. JAMA Netw. Open.

[47] Solch R.J., Aigbogun J.O., Voyiadjis A.G., Talkington G.M., Darensbourg R.M., O’Connell S., Maraganore D.M. (2022). Mediterranean diet adherence, gut microbiota, and Alzheimer’s or Parkinson’s disease risk: A systematic review. J. Neurol. Sci..

[48] Stadlbauer V., Engertsberger L., Komarova I., Feldbacher N., Leber B., Pichler G., Fink N., Scarpatetti M., Schippinger W., Schmidt R. (2020). Dysbiosis, gut barrier dysfunction and inflammation in dementia: A pilot study. BMC Geriatr..

[49] Tuigunov D., Sinyavskiy Y., Nurgozhin T., Zholdassova Z., Smagul G., Omarov Y., Omarova I. (2025). Precision Nutrition and Gut–Brain Axis Modulation in the Prevention of Neurodegenerative Diseases. Nutrients.

[50] Fattorusso A., Di Genova L., Dell’Isola G.B., Mencaroni E., Esposito S. (2019). Autism spectrum disorders and the gut microbiota. Nutrients.

[51] Hu E., Li Z., Li T., Yang X., Ding R., Jiang H., Wang Y. (2023). A novel microbial and hepatic biotransformation-integrated network pharmacology strategy explores the therapeutic mechanisms of bioactive herbal products in neurological diseases: The effects of Astragaloside IV on intracerebral hemorrhage as an example. Chin. Med..

[52] Zhang S., Wu Z., Zhang S., Ru Y., Wang Q., Tong H., Wu G. (2025). The intricate microbial-gut-brain axis in Alzheimer’s disease: A review of microbiota-targeted strategies. Food Funct..

[53] Liu H., Zhu K., Yang C. (2024). The intersection between tryptophan-kynurenine pathway metabolites and immune inflammation, hormones, and gut microbiota in perinatal depression. Actas Esp. Psiquiatr..

[54] Wen Y., Liu Q., Zeng H., Lyu L., He X., Zhang X., Xiao Y. (2025). Age-specific reference intervals for plasma amino acids and their associations with nutrient intake in the Chinese pediatric population. iMeta.

[55] Chen X., Chen C., Ma C., Kang W., Wu J., Fu X. (2025). Dendrobium officinale polysaccharide attenuates type 2 diabetes in mice model by modulating gut microbiota and alleviating intestinal mucosal barrier damage. Food Sci. Hum. Wellness.

[56] Li W.R., Li Y.K., Ren H., Guo Z., Xiao C.L., Luo J.Q. (2025). Lactobacillus reuteri attenuates methotrexate-induced liver injury via modulation of oxidative stress and inflammation through HO-1/GPX4 and NF-κB/NLRP3 pathways. Eur. J. Pharmacol..

